# Characterization of T cell responses against *Fusobacterium necrophorum* naturally-induced foot rot in dairy cows

**DOI:** 10.3389/fimmu.2025.1714457

**Published:** 2026-01-07

**Authors:** Yi Yang, Lulu Zhang, Chen Yu, Sheng Zhang, Chunmei Ye, Zhangping Yang, Zhipeng Zhang

**Affiliations:** 1 Jiangsu Co-Innovation Center for Prevention and Control of Important Animal Infectious Diseases and Zoonoses, College of Veterinary Medicine, Yangzhou University, Yangzhou, China; 2 College of Animal Science and Technology, Yangzhou University, Yangzhou, China; 3 Kang Yuan Dairy Co., LTD., Yangzhou University, Yangzhou, China

**Keywords:** cow, flow cytometry, foot rot, *Fusobacterium necrophorum*, T cell response

## Abstract

Bovine foot rot, an infectious disease caused by *Fusobacterium necrophorum*, leads to significant economic losses in the dairy farming. Research on bovine foot rot has primarily focused on the isolation and identification of the pathogen, as well as treatment methods. However, few studies have reported on the host’s immunological characteristics following infection. In this study, we employed an eight-color flow cytometry panel to characterize T-cell immune responses in dairy cows after infection by the *F. necrophorum* naturally-induced foot rot. We found that dairy cows with natural F. necrophorum-induced foot rot exhibited significantly increased percentages of NK cells, NKT cells, CD4^+^CD8^-^ (helper) T cells, and CD4^+^CD8^+^ (double-positive) T cells compared to healthy cattle, while the proportion of WC1^+^ γδ T cells remained unchanged. However, the frequency of CD44^High^ expressing cells was significantly elevated across all these T-cell subsets, suggesting their differential activation upon host infection with *F. necrophorum*. Compared with healthy cows, those with natural *F. necrophorum*-induced foot rot showed a significant increase in the percentages of effector memory T cells within NK and CD4^+^CD8^+^ T cell populations, and of central memory T cells within CD4^+^CD8^+^ and CD4^+^CD8^-^ T cells, respectively. Our results, for the first time, revealed a potential role of CD4^+^CD8^+^ T cells and NK cells in the defense of *F. necrophorum* naturally-induced foot rot in cows.

## 1 Introduction

Bovine foot rot (BFR) is one of the most common and prevalent infectious diseases in dairy herds (1). It primarily affects the interdigital skin and subcutaneous tissues of the hoof, leading to swelling in the interdigital space and resulting in suppurative necrosis of the dermis and keratin layer of the skin (2). The inflammation often extends from the interdigital skin to the coronary band, pastern, and fetlock joint, accompanied by systemic symptoms (3). This condition severely compromises dairy cow productivity and causes substantial economic losses to the cattle industry.

*F. necrophorum* is a strictly anaerobic, Gram-negative, non-motile, and pleomorphic bacterium belonging to the genus Fusobacterium. It is widely distributed in soil, sewage, and the microbiota of the oropharyngeal and gastrointestinal tracts of humans and animals (4). Due to its commensal nature, *F. necrophorum* is a notable opportunistic pathogen capable of causing Lemierre’s syndrome in humans, bovine foot rot, and other necrotizing infections (5, 6). The pathogenesis of *F. necrophorum* is associated with a variety of virulence factors that facilitate its survival, proliferation, and evasion of host immune defenses (7). Major virulence factors include lipopolysaccharide (LPS), hemolysin, adhesins, and leukotoxin (8, 9). Furthermore, *F. necrophorum* produces hydrogen sulfide and butyric acid, which promote tissue necrosis and create anaerobic conditions conducive to its growth. The bacterium’s ability to form biofilms protects it from immune clearance and antimicrobial therapy (10).

The growing prevalence of antimicrobial resistance among anaerobic bacteria poses significant challenges, making vaccine development a focused research area to overcome the limitations of antibiotic therapy in managing *F. necrophorum* infections in both human and veterinary medicine. Significant efforts have been devoted to the development of vaccines against *F. necrophorum*. For instance, immunodominant outer membrane proteins of *F. necrophorum* isolated from sheep with severe foot rot have been identified and proposed as candidates for recombinant protein-based subunit vaccines (11). In addition, computational biology approaches have been employed to design a multi-epitope vaccine targeting transmembrane proteins of *F. necrophorum*, aiming to elicit a robust immune response (12). However, no commercial vaccine against *F. necrophorum* is currently available on the market. Conventional vaccine evaluation studies have predominantly focused on antibody responses, while the cell-mediated immune mechanisms underlying the protection induced by these vaccines remain poorly understood. Therefore, characterizing the cell-mediated immune responses elicited by *F. necrophorum* infection may provide critical insights into protective immunity against this pathogen, thereby guiding the development of more effective and broad-spectrum vaccines.

Crucially, the nature of the T-cell response differs fundamentally between transient physiological challenges and persistent pathological conditions. Under physiological conditions, T-cell activation is a tightly regulated, transient process that resolves upon antigen clearance, maintaining immune homeostasis. In contrast, during chronic infections such as foot rot, the persistent presence of bacterial antigens like *F. necrophorum* can lead to sustained T-cell activation. This prolonged stimulation is a double-edged sword: while necessary to control the infection, it can also drive T cells towards a state of functional exhaustion or dysregulation, characterized by a progressive loss of effector functions and the expression of inhibitory receptors (13). Therefore, understanding the specific character of the T-cell response in a chronic setting like foot rot is not only vital for elucidating the immunopathology of the disease but also for identifying potential therapeutic targets to modulate an otherwise dysfunctional immune response.

This study aims to characterize the cell-mediated immune responses induced by natural *F. necrophorum* infection in cattle with foot rot, with a particular focus on T-cell activation and memory profiles following chronic infection. To our knowledge, this study provides the most comprehensive assessment to date of the cell-mediated immune response to *F. necrophorum*, offering valuable insights into the immune mechanisms underlying BFR and potentially broadening the understanding of immunity against other infectious diseases induced by this pathogen.

## 2 Materials and methods

### 2.1 Sampling strategy and sample collection

All experiments were conducted in accordance with the ethical guidelines for scientific research and were reviewed and approved by the Institutional Animal Care and Use Committee (IACUC) of Yangzhou University (Approval ID: 202503247). This study was conducted on a well-managed dairy farm in Jiangsu, China. All cows were sourced from a herd with documented BVD-free and IBR-vaccinated status, participating in ongoing Para TB control programs. Animals were clinically observed daily for systemic signs of disease. Inclusion criteria for the foot rot group required an acute, localized presentation of foot rot without evidence of concurrent illness. Control animals were free of any foot lesions and lameness, and were matched to the diseased group based on herd health management practices. A total of 20 cows were diagnosed with lameness by veterinary clinicians, of which 18 were further confirmed to have foot rot. The foot rot cases were characterized by painful interdigital lesions, necrotic margins, foot swelling, and swelling that was symmetric about the axial midline of the foot. The diseased animals were identified from within the general herd and, following diagnosis and sampling, were typically moved to a dedicated hospital pen for treatment and recovery. Regarding the pen size, the home pens were approximately 20 x 30 meters in size.

From each animal, one affected leg was selected for sampling. The diseased limb was restrained with a rope and elevated, after which the lesion was examined and superficially cleaned with water before being dried with clean paper towels. Local anesthesia was induced via subcutaneous injection of 3 ml lidocaine without bacteriostatic preservatives. A 4 mm punch biopsy tool was used to obtain skin biopsy specimens from the margin of the foot lesion. The skin biopsy samples were immediately transported to Yangzhou University for subsequent analysis. In addition to the cows affected by foot rot, we also selected 15 healthy cows that were closely matched in terms of age, parity, and body condition. The healthy control group (n=15) consisted of animals from the same herd with no history of lameness in the preceding 3 months. All control subjects underwent a thorough clinical examination to confirm the absence of any hoof lesions (Locomotion Score ≤ 2) and systemic illness. Blood samples were collected from the tail vein (coccygeal vein) using a sterile 21-gauge needle into 10 mL vacuum tubes containing EDTA as an anticoagulant. The sampling site on the tail was thoroughly disinfected with 70% ethanol. Following collection, samples were gently inverted 8–10 times for immediate mixing and were maintained at room temperature before processing for flow cytometry analysis within 4 hours of collection. To avoid confounding effects of antibiotic therapy, all samples were collected prior to the initiation of veterinary intervention.

### 2.2 16S rDNA gene sequencing and analysis

Genomic DNA was extracted from approximately 25 mg of skin biopsy tissue using the Tiangen Biotech DNA Extraction Kit (Beijing, China), following the manufacturer’s instructions for Gram-negative bacteria with minor modifications. Briefly, the tissue sample was mechanically lysed in a lysis buffer containing Proteinase K and incubated at 56 °C for 12 hours until complete digestion. After incubation, the lysate was centrifuged, and the supernatant was transferred to a new microcentrifuge tube. Binding buffer was added, and the mixture was loaded onto a silica membrane column. The column was washed twice with the provided wash buffers to remove impurities. Finally, the genomic DNA was eluted in 50 μL of pre-heated (70 °C) Elution Buffer and stored at -20 °C until further analysis.

The hypervariable V3-V4 region of the bacterial 16S rRNA gene was amplified by PCR using the universal primers 27 F (5’-AGAGTTTGATCCTGGCTCAG-3’) and 1492R (5’-GGTTACCTTGTTACGACTT-3’) (14). The 25 μL PCR reaction mixture contained 2.5 μL of 10x PCR Buffer, 1 μL of each primer (10 μM), 2 μL of dNTP Mix (2.5 mM each), 0.5 μL of Taq DNA Polymerase, and 50 ng of template DNA. The amplification was performed under the following conditions: initial denaturation at 95 °C for 5 min; followed by 30 cycles of denaturation at 95 °C for 30 s, annealing at 55 °C for 30 s, and extension at 72 °C for 45 s; with a final extension at 72 °C for 7 min. The PCR products were visualized on a 1.5% agarose gel to confirm successful amplification of the ~460 bp target fragment.

The confirmed PCR products were purified and sent to a commercial laboratory (Qingke, Shanghai, China) for paired-end sequencing on an Illumina MiSeq platform. Bacterial species were identified by aligning the assembled sequences with reference databases using BLASTN. Only cows with foot rot lesions that were identified as being mono-infected with Fusobacterium necrophorum were ultimately included in the experimental group.

### 2.3 The isolation of PBMCs in blood

Peripheral blood mononuclear cells (PBMCs) were isolated from all anticoagulated blood samples by density gradient centrifugation (650 × g for 35 minutes) using lymphocyte separation medium (Tianjin Taipu Biological, China). The purified PBMCs were resuspended in FACS buffer (0.5% FBS in PBS) and adjusted to a concentration of 2 × 10^6^ cells per sample for phenotypic analysis of T cells (15).

### 2.4 Flow cytometry

**Figure 1:**
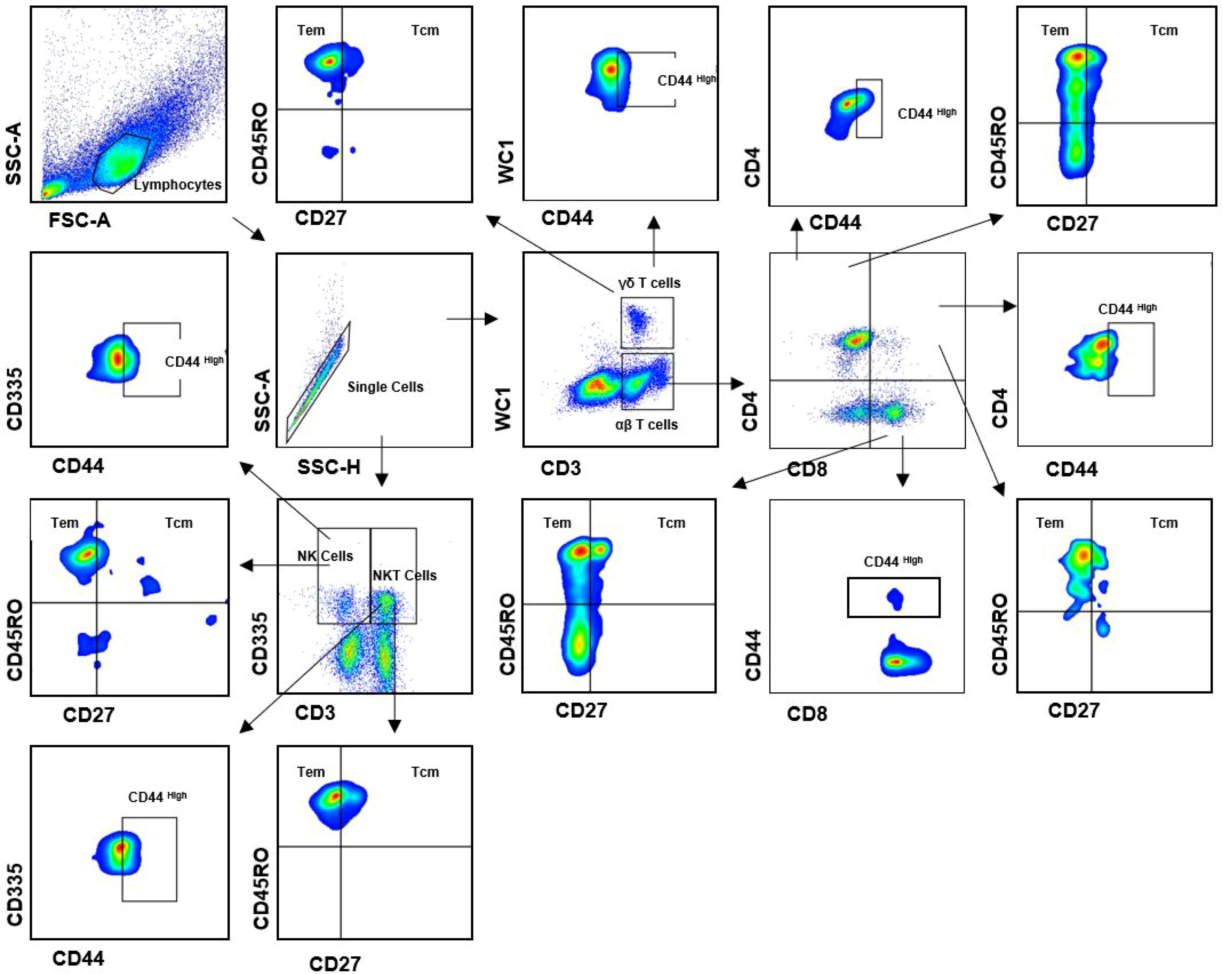
Gating strategies to define bovine T cell subsets. Mononuclear cells were isolated from PBMCs of cows and surface stained with antibody cocktails (CD3, WC1, CD335, CD4, CD8, CD44, CD27 and CD45RO). The lymphocytes are chosen with FSC versus SSC and then singlets are gated using SSC-A and SSC-H. By outputting CD3 versus WC1, CD3^+^WC1^+^ (γδ T cells), CD3^+^WC1^-^ are identified, respectively. By outputting CD3 versus CD335, CD3^+^CD335^+^ (NK T cells), CD335^+^CD3^-^ (NK cells) are identified, respectively. CD3^+^WC1^-^ T cells contain CD4^+^CD8^-^, CD4^-^CD8^+^ and CD4^+^CD8^+^ T cells. Both CD4^+^CD8^-^, CD4^-^CD8^+^ T cells can be further subdivided into CD44^+^ subsets, respectively. The γδ T cells, NK T cells, NK cells, CD4^+^CD8^-^, CD4^-^CD8^+^ and CD4^+^CD8^+^ T cells contains CD44^+^ γδ T cells, Tcm (CD45RO^+^CD27^+^) and Tem (CD45RO^+^CD27^-^).

Flow cytometry was performed to compare the relative abundance, activation state (based on CD44 expression), and memory differentiation of key lymphocyte populations between healthy cows and those with foot rot. PBMCs were stained in V-bottom plates and incubated with 1% bovine serum at 37°C for 20 minutes to block Fc receptors. Secondary antibodies and monoclonal antibodies (mAbs) used for cell surface staining are listed in **Table 1**. Cells were then centrifuged at 1600 rpm for 5 minutes at 4°C and washed with FACS buffer. A minimum of 1×10^5^ cells were collected for flow cytometric analysis. The gating strategy was based on fluorescence-minus-one (FMO) controls. The gating strategy is illustrated in **Figure 1**. Data were analyzed using FlowJo software (BD Biosciences, San Jose, USA) and further processed with GraphPad Prism 9.4 software (GraphPad, San Diego, USA) for statistical calculations and graphical representation.

**Table 1:** Antibodies used for flow cytometry in this study.

Marker	Clone	Isotype	Conjugate	Labeling strategy	Labeling strategy
CD3	MM1A	IgG_1_	PerCP-Cy5.5	Secondary antibody	Kingfisher
WC1	CC15	IgG2a	FITC	Direct conjugate	Bio-rad
CD4	CC8	IgG2a	AlexaFluor647	Direct conjugate	Bio-rad
CD8	CC63	IgG2a	PE-Cyanine7	Secondary antibody	Bio-rad
CD44	IMC	IgG2b, κ	PrestoBlue	Direct conjugate	BioLegend
CD335	AKS1	IgG1	PE	Direct conjugate	Bio-rad
CD45RO	IL-A116	IgG3	PE-Texas Red	Secondary antibody	Kingfisher
CD27	M-T271	IgG1, κ	BrilliantViolet510	Direct conjugate	BioLegend

### 2.5 Statistical analyses

All statistical analyses were performed using GraphPad Prism 9.4 software (GraphPad, San Diego, USA). For comparisons among more than two groups, one-way ANOVA followed by a *post-hoc* multiple comparisons test was applied; for comparisons between two groups, an unpaired t-test was used. Data are presented as mean ± standard deviation. A p-value of less than 0.05 was considered statistically significant (*P < 0.05, **P < 0.01, ***P < 0.001).

## 3 Results

### 3.1 Identification of the causative pathogen from skin biopsy samples confirmed *F. necrophorum* in cows with foot rot

Microbiological analysis of bacterial DNA from skin biopsy samples of 18 cows with foot rot revealed that 15 animals (83.3%) were mono-infected with *F. necrophorum*, indicating it as the sole causative agent. The remaining three cows showed mixed infections, involving either *Bacillus cereus* or a rare species of *Comamonas kerstersii* (**Table 2**). Only the 15 cows with exclusive F. necrophorum infection were included in subsequent experiments. All clinical profiles are also summarized in **Table 2**.

**Table 2:** The case report provided by an occupational veterinarian.

# Cow	Age/month	Lactation days	BCS	Diagnostic result	Diagnosis date	Treatment date	Recovery date	Treatment protocols	Pathogenic bacteria
18278	57	98	4	Foot rot	2022/12/13	2022/12/15	2023/1/8	All cows: combined penicillin, metronidazole, and clindamycin frequently administered.	Fusobacterium necrophorum
18018	58.8	93	5	Foot rot	2022/12/7	2022/12/10	2022/12/23		
18494	56.8	96	4	Foot rot	2022/12/9	2022/12/13	2023/1/2		
18441	57.6	99	5	Foot rot	2022/11/13	2022/11/17	2022/12/30		
18488	59.3	94	4	Foot rot	2022/12/01	2022/12/10	2023/1/9		
18654	59.8	91	5	Foot rot	2022/12/02	2022/12/05	2023/1/15		
18675	61.5	89	5	Foot rot	2022/12/06	2022/12/08	2023/1/16		
18679	62.7	93	5	Foot rot	2022/12/08	2022/12/12	2023/1/10		
18685	60.2	94	4	Foot rot	2022/12/07	2022/12/10	2023/1/12		
18247	59.4	92	5	Foot rot	2022/12/04	2022/12/06	2023/1/10		
18141	55.9	90	4	Foot rot	2022/12/09	2022/12/12	2023/1/11		
18144	59.9	88	5	Foot rot	2022/12/04	2022/12/06	2023/1/6		
18213	56.2	91	4	Foot rot	2022/11/08	2022/11/10	2022/12/19		
18248	56.1	86	5	Foot rot	2022/11/28	2022/11/30	2022/12/14		
18254	61.4	92	4	Foot rot	2022/12/01	2022/12/04	2022/12/17		
18255	60.1	90	4	Foot rot	2022/11/08	2022/11/10	2022/12/19		Fusobacterium necrophorum, Comamonas kerstersii
18311	59.5	91	4	Foot rot	2022/11/28	2022/11/30	2022/12/14		Fusobacterium necrophorum, Comamonas kerstersii
18329	61.3	89	5	Foot rot	2022/12/01	2022/12/04	2022/1/17		Fusobacterium necrophorum, Bacillus cereus

Veterinarian: Ming Wang Date: 2023/2/1 Address: Yonghao Dairy Farm, Suining County, Xuzhou City, Jiangsu Province, China.

### 3.2 Infection with *F. necrophorum* resulted in a higher percentage of peripheral blood NK cells, NKT cells, CD4^+^CD8^-^ and CD4^+^CD8^+^ T cells

**Figure 2:**
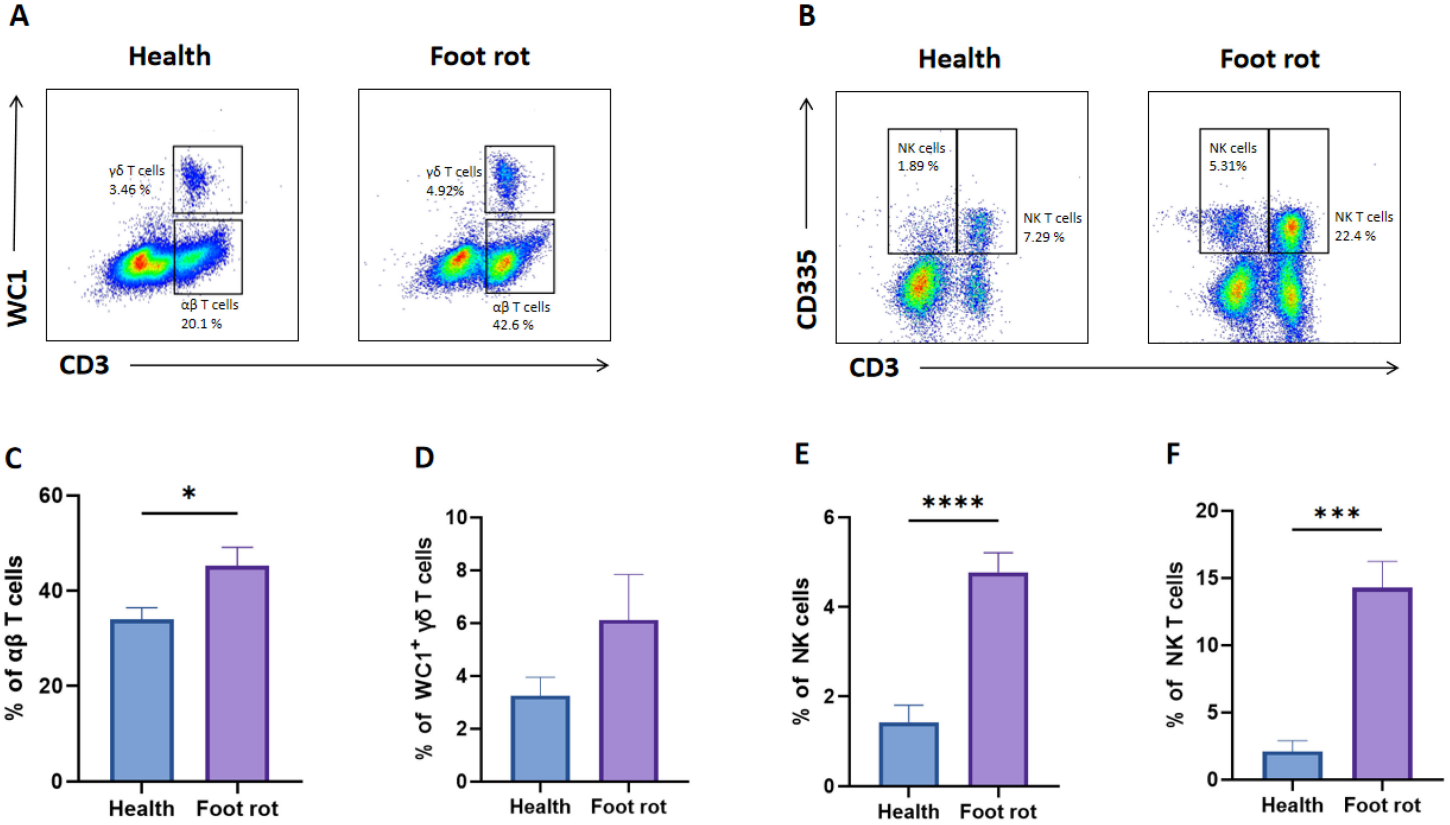
The distribution of αβ, γδ, NK T cells and NK cells in the peripheral blood of healthy and *F*. *necrophorum* naturally-induced foot rot in dairy cows. **(A)** Representative dot plots of WC1^+^ γδ T cells and WC1^−^ CD3^+^ αβ T cells from two groups. **(B)** Representative dot plots of NK cells (CD335^+^CD3^-^) and NK T cells (CD335^+^CD3^+^) from two groups. The percentage differences of αβ **(C)** and γδ T cells **(D)**, NK cells **(E)** and NK T cells **(F)** between healthy and foot rot dairy cows. A p-value of less than 0.05 was considered statistically significant (*P < 0.05, ***P < 0.001, ****P < 0.0001).

The changes in bovine peripheral T-cell subsets following Fusobacterium necrophorum infection have not been well characterized (16). In this study, we used flow cytometry to analyze alterations in T-cell populations in dairy cows with natural *F. necrophorum*-induced foot rot. As shown in representative flow plots (**Figures 2A, B**), infection with *F. necrophorum* resulted in a significant increase in the percentages of αβ T cells, NK cells (CD335^+^CD3^−^), and NKT cells (CD335^+^CD3^+^) compared with healthy cows, whereas the proportion of WC1^+^ γδ T cells remained unchanged (**Figures 2C–F**). This suggests that the αβ T cell compartment itself is undergoing a true expansion, which increases its share of the total lymphocyte pool, while the proportions of both B cells and γδ T cells are diluted accordingly.

**Figure 3:**
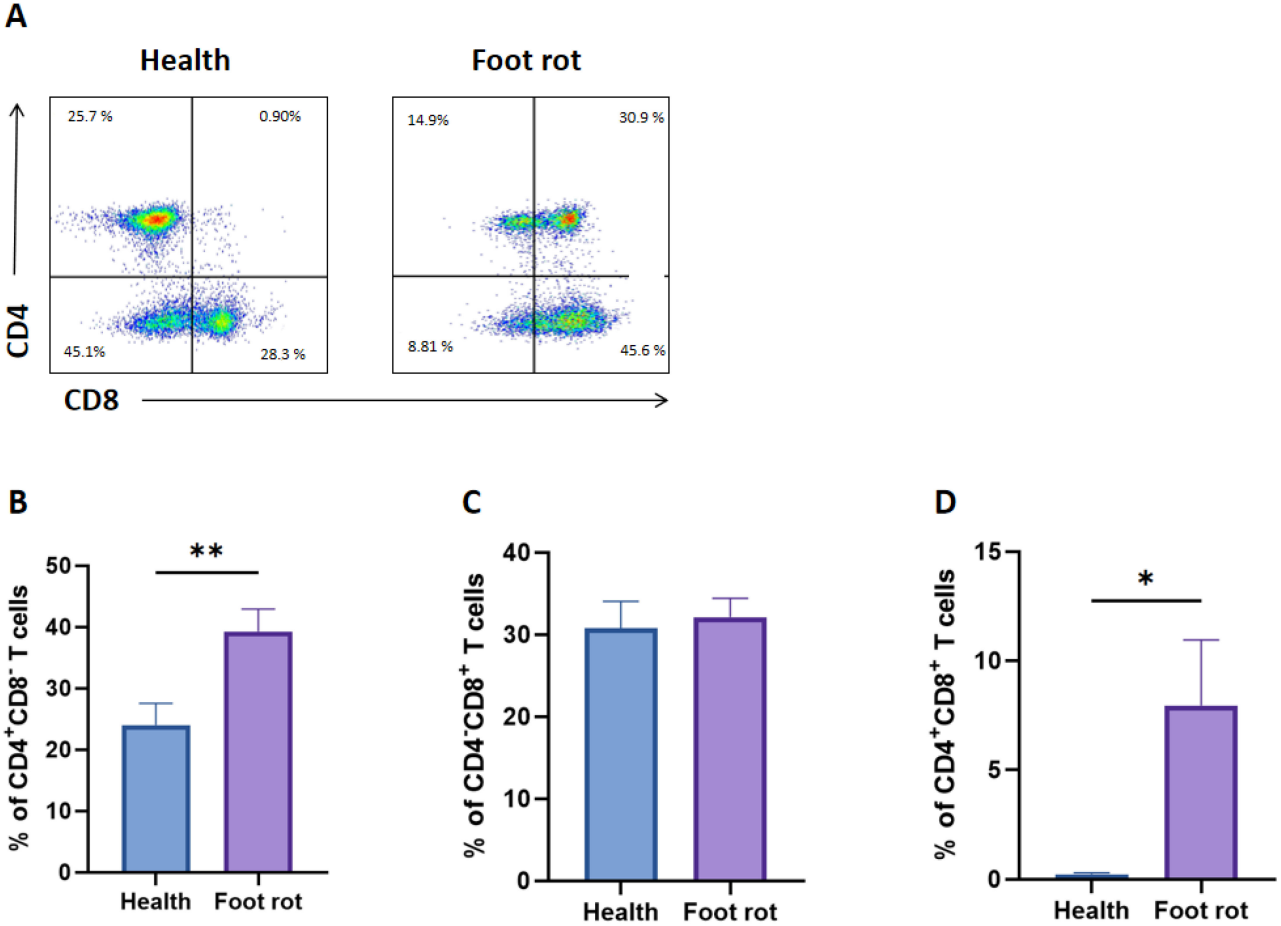
The distribution of CD4^+^CD8^-^, CD4^-^CD8^+^and CD4^+^CD8^+^ T cells in T cells of healthy and *F*. *necrophorum* naturally-induced foot rot in dairy cows. **(A)** Representative dot plots of CD4^+^CD8^-^, CD4^-^CD8^+^ and CD4^+^CD8^+^ T cells from two groups. The percentage differences of CD4^+^CD8^-^**(B)**, CD4^-^CD8^+^**(C)** and CD4^+^CD8^+^**(D)** T cells between healthy and foot rot dairy cows. A p-value of less than 0.05 was considered statistically significant (*P < 0.05, **P < 0.01).

Furthermore, it remained unclear whether the proportion of different αβ T-cell subsets differed between the two groups. Therefore, we distinguished three T-cell subsets (CD4^+^CD8^−^, CD4^−^CD8^+^, and CD4^+^CD8^+^) in the αβ T cells (**Figure 3A**). As illustrated in **Figures 3B–D**, the percentage of CD4^+^CD8^−^ and CD4^+^CD8^+^ T cells in the Foot rot groups increased significantly to healthy cows. However, CD4^−^CD8^+^ has no significant change.

### 3.3 *F. necrophorum* infection was associated with enhanced activation levels in all T-cell subsets examined from cows exhibiting foot rot

**Figure 4:**
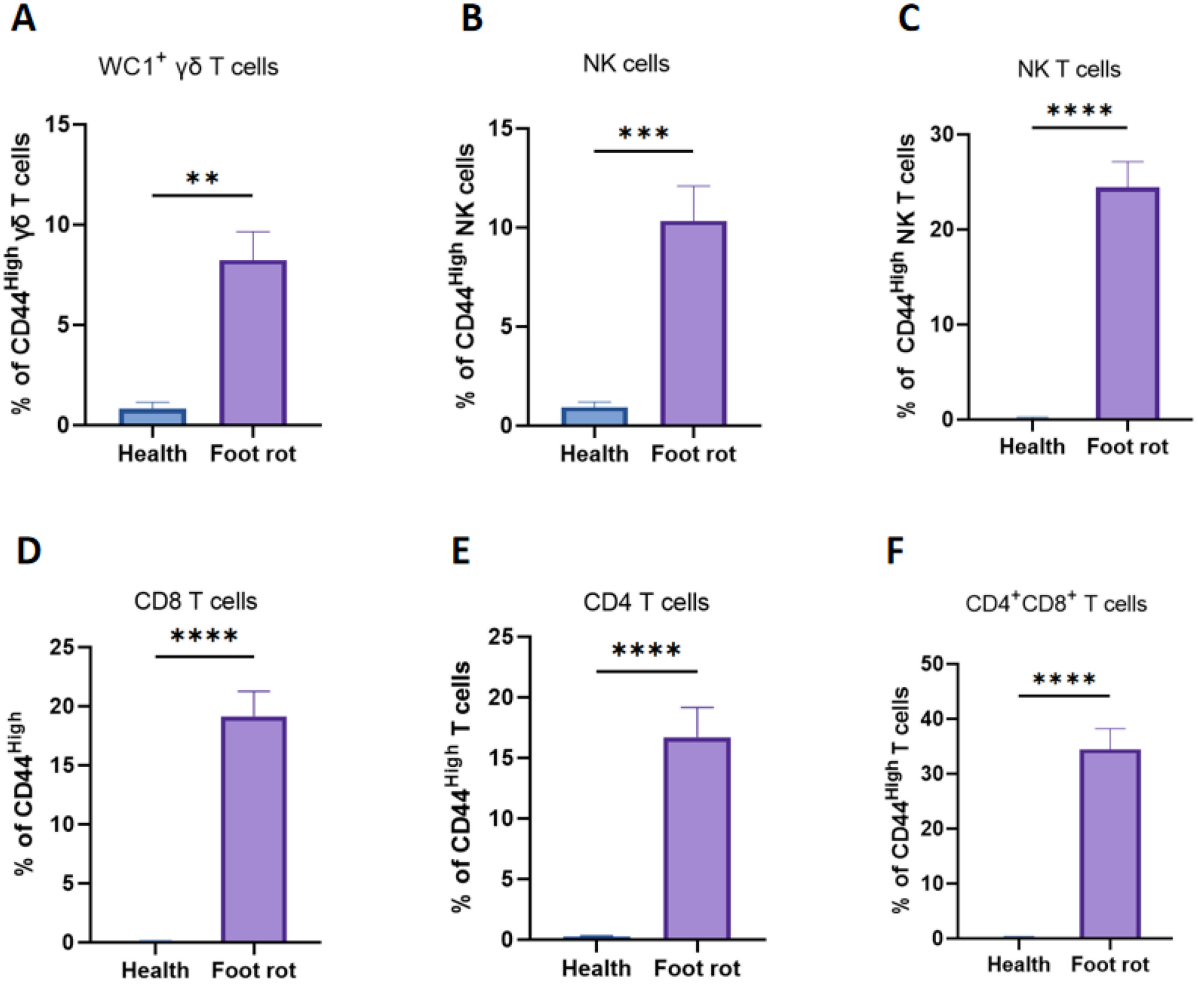
Difference of CD44 ^High^ expression in different subsets of T-cell subsets (WC1^+^ γδ, NK, CD8^+^, CD4^+,^CD4^+^CD8^+^) and NK cells of healthy and *F*. *necrophorum* naturally-induced foot rot in cows. The histograms show different CD44 ^High^ percentages on WC1^+^ γδ **(A)**, NK **(C)**, CD8^+^**(D)**, CD4^+^**(E)**, CD4^+^CD8^+^**(F)** T cells and NK cells **(B)** between the healthy and foot rot cows. A p-value of less than 0.05 was considered statistically significant (**P < 0.01, ***P < 0.001, ****P < 0.0001).

To determine the activated phenotype of T cells between healthy and foot rot cows, infection-experienced T cells were identified based on high expression of CD44. Following the infection, WC1^+^ γδ, NK, CD4^-^CD8^+^, CD4^+^CD8^-^, CD4^+^CD8^+^ T cells and NK cells all exhibited a significant increased percentage of the CD44 ^High^ phenotype (**Figure 4**).

### 3.4 Memory phenotype in T-Cell subsets from cows with *F. necrophorum*-induced foot rot

**Figure 5:**
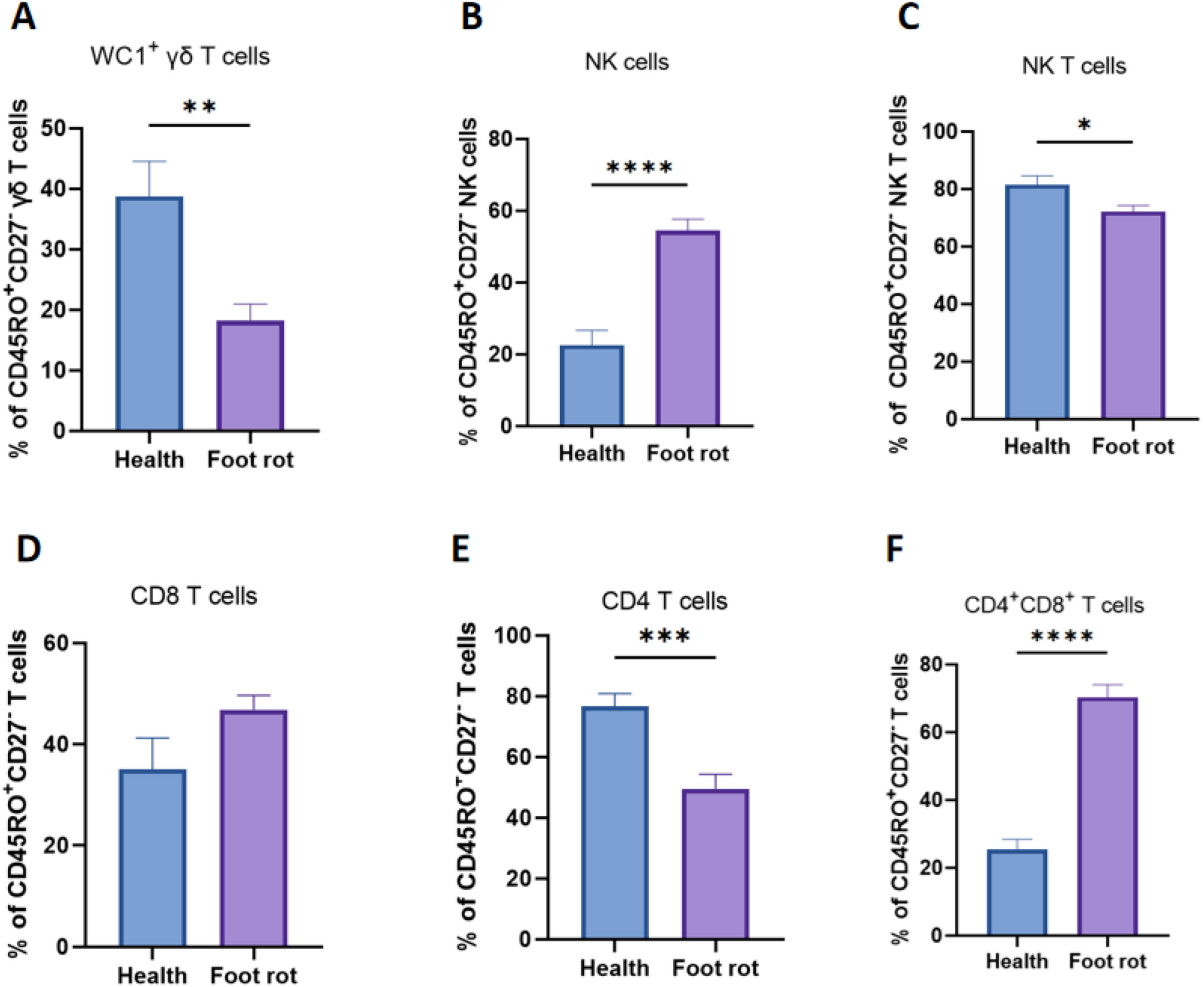
Dynamic changes in cow effector memory T cells after natural *F*. *necrophorum* infection in cattle with foot rot. The percentages of CD27^+^CD45RO^−^ T cells (Tem) were compared in WC1^+^ γδ **(A)**, NK **(C)**, CD8^+^**(D)**, CD4^+^, **(E)**, CD4^+^CD8^+^**(F)** T cells and NK cells **(B)** in heathy and foot rot cows. A p-value of less than 0.05 was considered statistically significant (*P < 0.05, **P < 0.01, ***P < 0.001, ****P < 0.0001).

The effector memory T cells are defined as CD45RO^+^CD27^−^, and central memory T cells are defined as CD45RO^+^CD27^+^ in this study (17). As proven in **Figure 5**, the percentage of effector memory T cells in NK cells and CD4^+^CD8^+^ T cells increased significantly after being infected by *F. necrophorum* naturally-induced foot rot compared with healthy dairy cows. However, in WC1^+^ γδ, NK and CD4 T cells, the percentage of effector memory T cells was significantly reduced after being infected. There existed no crucial distinction in the percentage of CD8 T cells with central memory phenotype before and after being infected.

**Figure 6:**
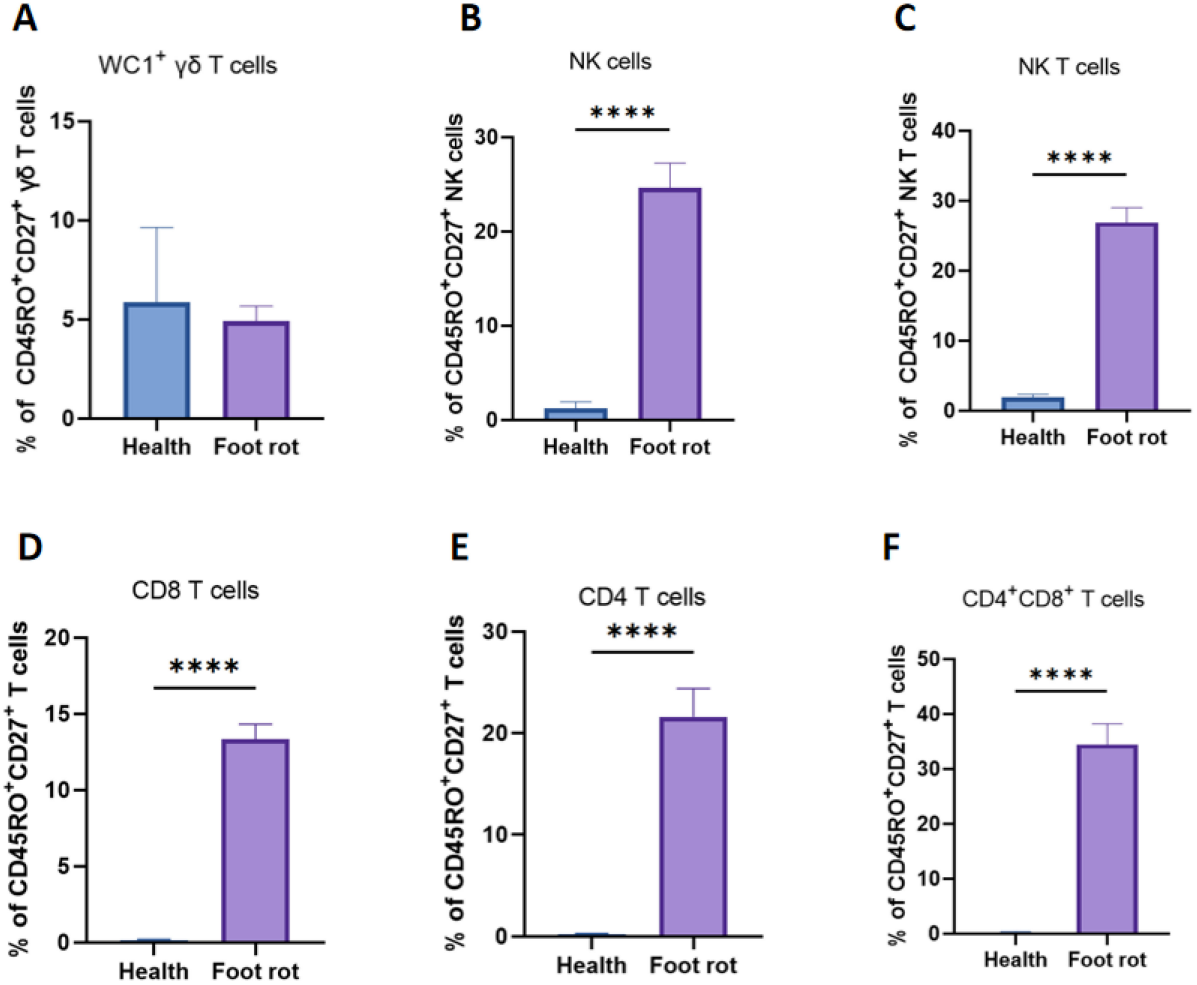
Dynamic changes in cow central memory T cells after natural *(F) necrophorum* infection in cattle with foot rot. The percentages of CD27 ^+^ CD45RO ^+^ T cells (Tcm) were compared in WC1^+^ γδ **(A)**, NK **(C)**, CD8^+^**(D)**, CD4^+^, **(E)**, CD4^+^CD8^+^**(F)** T cells and NK cells **(B)** in heathy and foot rot cows. A p-value of less than 0.05 was considered statistically significant (****P < 0.0001).

The dynamic changes in central memory cells (**Figure 6**) in NK cells, CD8 and CD4 T cells were different. In NK cells, CD8 and CD4 T cells, the percentage of central memory T cells was significantly increased after being infected compared with healthy dairy cows. There existed no crucial distinction in the percentage of WC1^+^ γδ T cells with naïve phenotype before and after being infected.

## 4 Discussion

Historically, the evaluation of BFR has primarily focused on the identification and characterization of various pathogenic microorganisms, while overlooking the critical role of T cell-mediated cellular immunity in both the detection and treatment of the disease (18). Therefore, this study investigates the importance of a robust T-cell response in clearing pathogens and establishing effective memory responses in BFR from the perspective of T cell-mediated immunity. In this experiment, a new eight-color flow cytometry was employed to assess the cellular immune responses in dairy cows, with a specific focus on T-cell immune responses to BFR caused by *F. necrophorum*. Although previous studies have established an eight-color flow cytometry panel for bovine T-cell phenotyping (19), the functional characteristics of T-cell subsets in both healthy and infected cattle remain undefined. In contrast, the functional attributes of the cell subsets identified in this study have been partially validated, facilitating a more reliable assessment of T-cell mediated immune responses during BFR (17, 20).

To explore the mechanisms of T cell-mediated immunity in affected cattle, a comparative design was implemented, grouping animals into healthy controls and those suffering from BFR induced by *F. necrophorum.* It should be noted that the healthy control animals were defined by the absence of clinical disease and were not microbiologically tested for subclinical carriage of pathogens such as *F. necrophorum*. Therefore, the observed immunological differences are interpreted as being associated with the clinical manifestation of foot rot. Based on this grouping, we analyzed the distribution and dynamic changes of various T-cell subsets in peripheral blood following infection. It was observed that BFR caused by *F. necrophorum* led to a significant increase in the proportions of several T-cell subpopulations, including αβ (WC1^−^CD3^+^) T cells, NK (CD335^+^CD3^−^) cells, and NK T (CD335^+^CD3^+^) cells. While the present study reveals this shift in lymphocyte subsets presented as percentages, it is important to acknowledge its inherent limitation: the absence of absolute cell count data. Interpretation of proportional data alone can be ambiguous. However, a critical observation that the proportion of WC1^+^ γδ T cells showed little change. This may be attributed to their involvement in earlier immune stages, whereas the infected cows selected for this study were chronically infected, with infection durations exceeding 15 days—well beyond the early phase—thus likely missing the window for detecting dynamic changes in this subset (21). This argues against a general contraction of the B cell lineage and instead points to a true expansion of the αβ T cell population. Future studies will establish a more frequent and precise monitoring system.

Furthermore, using CD4 and CD8 markers, we analyzed changes in αβ T cell subpopulations between healthy and diseased dairy cows. Significant increases were observed in both CD4^+^CD8^−^ and CD4^+^CD8^+^ T cell populations, while CD4^−^CD8^+^ T cells showed unnotable change. This suggests that CD8^+^ T cells may not play a direct role in BFR, though further investigation is required to confirm this. CD8^+^ T cells are crucial components of the adaptive immune system, orchestrating cytotoxic responses. Their relative abundance is closely associated with bolstered immune defense, particularly when tissue injury or infection is most likely to occur (22). Traditionally, CD4^+^CD8^+^ double-positive (DP) T cells were considered a developmental stage in the thymus, with few mature DP T cells found in peripheral blood (23). However, studies over recent decades have unexpectedly identified mature CD4^+^CD8^+^ T cells in the peripheral blood of humans, dogs, and pigs (24). In bovine mastitis research, CD4^+^CD8^+^ DP T cells have been shown to participate in various immune functions, influencing cytokine production and thereby modulating immune responses (20).

Similar to integrin expression, CD44 expression is upregulated following lymphocyte activation, promoting migration through the extracellular matrix via interactions with hyaluronic acid and fibrinogen (25). To clarify whether various T-cell subsets are indeed involved in the immune response to *F. necrophorum*-induced BFR, we used high CD44 expression as an indicator of activation across different T cell populations. Results showed that WC1^+^ γδ, NK, CD8^+^, CD4^+^, CD4^+^CD8^+^ T cells and NK cells, were all activated. An interesting observation emerged when correlating activation with expansion: CD8^+^ T cells were activated but did not expand, indicating a potential participatory role in the immune response that warrants further investigation into the underlying mechanisms.

Memory T cells are capable of mounting rapid and robust immune responses, which is a hallmark of host defense (26). In this study, effector memory T cells (TEM) were defined as CD45RO^+^CD27^−^, and central memory T cells (TCM) as CD45RO^+^CD27^+^. TEM cells are a crucial subset in T-cell immune responses. An increase in TCM enhances immune defense, sustains long-term protection, and participates in chronic inflammation and autoimmune diseases (27). They also serve as indicators of disease prognosis and treatment response. A decrease in TEM may impair the maintenance of immunological memory, suggest immune dysfunction or dysregulation, and potentially compromise vaccine-induced protection (28). In *F. necrophorum*-induced BFR, the proportions of NK cells and CD4^+^CD8^+^ T cells within the TEM compartment increased significantly, whereas WC1^+^ γδ, NK and CD4^+^ T cells decreased markedly.

TCM cells also play an indispensable role in the immune system, primarily by enhancing immunological memory (29). In BFR caused by *F. necrophorum*, significant increases were observed in the proportions of NK cells, NK, CD8^+^, CD4^+^ and CD4^+^CD8^+^ T cells within the TCM compartment. TCM cells exhibit long-term survival and self-renewal capabilities. An increase in TCM numbers implies an expanded immune memory repertoire, enabling longer-lasting antigen-specific recall (30). Upon re-exposure to the same antigen, TCM cells can rapidly activate and differentiate into effector T cells, mounting a faster and more effective immune response, thereby improving protection against reinfection (31). Moreover, TCM abundance is a key indicator of vaccine success, as vaccination aims to induce immunological memory (32).

In conclusion, this study provides a novel research direction for detecting *F. necrophorum*-induced BFR by monitoring changes in T-cell subsets. We observed significant increases in the proportions of αβ, γδ, NK T cells and NK cells in the peripheral blood of infected cows, accompanied by synchronous changes in CD44 expression. Surprisingly, CD4^−^CD8^+^ T cells showed no significant change before and after infection. However, the correlation between these findings and challenge protection remains to be fully elucidated. Overall, this study emphasizes the importance of T-cell responses in evaluating *F. necrophorum-*induced BFR in dairy cows, and the findings contribute to a deeper understanding of the immune mechanisms invoked by *F. necrophorum* infection.

## Data Availability

The original contributions presented in the study are included in the article/supplementary material. Further inquiries can be directed to the corresponding author.
